# Deep Convolutional Neural Networks With Ensemble Learning and Generative Adversarial Networks for Alzheimer’s Disease Image Data Classification

**DOI:** 10.3389/fnagi.2021.720226

**Published:** 2021-08-17

**Authors:** Robert Logan, Brian G. Williams, Maria Ferreira da Silva, Akash Indani, Nicolas Schcolnicov, Anjali Ganguly, Sean J. Miller

**Affiliations:** ^1^Pluripotent Diagnostics Corp. (PDx), Molecular Medicine Research Institute, Sunnyvale, CA, United States; ^2^Eastern Nazarene College, Quincy, MA, United States

**Keywords:** deep convolutional neural network, magnetic resonance imaging, Alzheimer’s disease, positron emission tomography, ensemble learning, generative adversarial network

## Abstract

Recent advancements in deep learning (DL) have made possible new methodologies for analyzing massive datasets with intriguing implications in healthcare. Convolutional neural networks (CNN), which have proven to be successful supervised algorithms for classifying imaging data, are of particular interest in the neuroscience community for their utility in the classification of Alzheimer’s disease (AD). AD is the leading cause of dementia in the aging population. There remains a critical unmet need for early detection of AD pathogenesis based on non-invasive neuroimaging techniques, such as magnetic resonance imaging (MRI) and positron emission tomography (PET). In this comprehensive review, we explore potential interdisciplinary approaches for early detection and provide insight into recent advances on AD classification using 3D CNN architectures for multi-modal PET/MRI data. We also consider the application of generative adversarial networks (GANs) to overcome pitfalls associated with limited data. Finally, we discuss increasing the robustness of CNNs by combining them with ensemble learning (EL).

## Introduction

The primary risk factor for developing Alzheimer’s disease (AD) is advanced age (Hebert et al., 2010). The global prevalence of AD is expected to double from the current burden of 50 million to 100 million by 2050 (2021 Alzheimer’s disease facts and figures, 2021). The United States alone has spent over $300 billion on AD treatments in 2020, excluding an estimated $256.7 billion in unpaid AD-related care (2021 Alzheimer’s disease facts and figures, 2021). AD is a complex multifactorial neurodegenerative disease with no cure. As such, there is a critical need for the development of viable treatment options. Widely varying pathology and patient heterogeneity contribute to our overall lack of understanding of etiology and underlying causes of neurodegeneration.

Currently, the gold standard for establishing a diagnosis and a prognosis of neurodegenerative diseases, such as AD is based on clinical assessment of symptoms and their severity. However, early disease detection before clinical symptom onset is crucial for disease management and timely therapeutic intervention. Research shows that medical imaging techniques, such as MRI and PET scans can detect structural and functional changes in the brains of patients in the early stages of AD (Franke et al., 2020). Machine learning approaches can be a quick and robust way to interpret medical imaging and aid in early diagnosis of AD.

Convolutional neural networks (CNNs) are deep multilayer artificial neural networks (Albawi et al., 2017). CNNs contain convolution layers that allow the model to extract feature maps obtained by the product of the input and a learned kernel, which are used to detect patterns, such as edges and local structures. The ability for quick feature extraction makes them highly efficient in pattern recognition in image data analysis. Furthermore, they have been demonstrated to be highly accurate in image classification, including medical imaging (Li et al., 2014; Setio et al., 2016; Wang et al., 2018). In image segmentation for organ and body part discrimination, CNNs outperformed other algorithms, such as logistic regression and support vector machines that do not have intrinsic feature extraction capabilities (Yan et al., 2016). For example, computer-aided diagnosis (CAD) systems based on CNNs have been successfully employed to detect lung cancer and pneumonia from X-ray imaging and macular degeneration from optical coherence tomography (OCT) (Kermany et al., 2018). For AD, an approach based on dual-tree complex wavelet transform for feature extraction followed by classification by a feedforward neural network was recently proposed (Jha et al., 2017). CNN architectures, such as GoogLeNet and ResNet have achieved strong results in distinguishing healthy from AD and mild cognitive impairment (MCI) brains using MRI imaging data (Prakash et al., 2019).

In addition to CNNs, ensemble learning (EL) has been shown to be valuable in medical imaging analysis. The frequent limited availability and the common 3D nature of medical imaging data can present a challenge when training classifiers (Dong et al., 2020). EL can be leveraged to overcome these limitations through combining multiple trained models. Therefore, EL can be used for classification using heterogeneous datasets (i.e., images from different imaging sources). Once individual classifiers are trained on each subset, they are then combined (Parikh and Polikar, 2007). EL with bootstrapping is especially helpful when relevant medical imaging data availability is limited (Bauer and Kohavi, 1999; Ganaie et al., 2021). Alternatively, limited data is commonly augmented by rotating and flipping existing images around an axis as well as using zooming functions.

Using generative adversarial networks (GANs) is another popular approach in augmenting imaging data. GANs create new data that compete with a discriminative whose role is to classify these new data as real or synthetic (Goodfellow et al., 2014). Generative networks that outcompete discriminative models can be leveraged to generate artificial data based on the underlying structure of real data (Wu et al., 2017). In the field of medical imaging, GANs have been successfully used for MRI and CT reconstruction and unconditional synthesis (Wolterink et al., 2017; Yi et al., 2019).

A commonly used resource in studying AD imaging data with the application of deep learning is the Alzheimer’s Disease Neuroimaging Initiative (ADNI) reference dataset. This neuroimaging database includes data from AD, mild cognitive impairment (MCI), and healthy individuals (Petersen et al., 2010). It consists of over 50,000 patient images and is often used to test the performance of models in AD image classification. In this review, we discuss recent exciting advances in the deep learning analysis of neuroimaging for AD patient diagnostics.

## Background

### Multilayer Perceptron Neural Networks

Neuronal networks are assumed to function in a hierarchical manner when detecting and interpreting a visual image. The first functional layer of neurons might be responsible for detecting the presence and location of edges within an image. The second functional neuronal layer then identifies individual features of the image. Finally, a third layer of neurons assimilate all features to recognize the image as a whole and assign meaning to the image in a broad context. Warren McCulloch and Walter Pitts famously posited 10 theorems in 1943 that was the first computational description of neuronal behavior (McCulloch and Pitts, 1943). Under the McCulloch-Pitts model, the artificial neuron is the smallest functional unit in a neural network. A McCulloch-Pitts artificial neuron receives one or more non-weighted Boolean inputs *x*_1_,…,*x*_*n*_*ε*{0,1} passed through a simple aggregation function with output $\hat{y}$. The inputs serve in either an excitatory or inhibitory manner. As with a biological neuron, a threshold needs to be surpassed in order to undergo an “all-or-none firing” to propagate messages. Typically, several excitatory inputs are needed to surpass the threshold. Additionally, since inputs are Boolean, a solitary inhibitory input can exert a stronger influence over whether or not a McCulloch-Pitts neuron fires than a solitary excitatory input when several synaptic inputs are involved. Indeed, inhibitory inputs hold veto power over excitatory inputs. Finally, if neurons receive no inhibitory input and the excitatory input exceeds the determined threshold, all neurons that meet these criteria simultaneously generate an output.

Fifteen years later, Frank Rosenblatt published his model for neuronal storage and organization of information. He coined the term “perceptron” to describe his version of the artificial neuron (Rosenblatt, 1958). Minsky and Papert (1969, 2017) further developed the perceptron model. The perceptron model was developed to more closely resemble the higher functions of the brain than the McCulloch-Pitts model, especially in terms of supervised learning. In contrast to the McCulloch-Pitts artificial neuron, which can only receive Boolean inputs, a perceptron can receive weighted inputs where certain inputs can exert more influence than others. Furthermore, inputs can be both excitatory and inhibitory, without the absolute veto power of inhibitory inputs as seen in the McCulloch-Pitts model. Additionally, the perceptron output function is a binary linear classifier that yields [−1, 1] rather than the McColloch-Pitts output of [0, 1] due to a change in the activation function. There is no perceptron solution for data that cannot be separated in a linear manner. Therefore, under the perceptron model, the XOR logical function cannot be solved.

**FIGURE 1:**
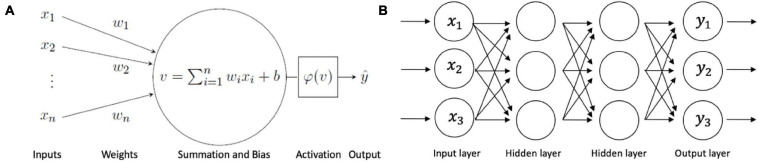
Multilayer perceptron neural network. **(A)** The perceptron artificial neuron. Input datapoints are represented as *x*_1_,*x*_*n*_, with synaptic weights *w*_1_,*w*_*n*_ and bias *b*. The local induced field *v* is passed through activation function φ to generate output $\hat{y}$. **(B)** Simplified example of a multilayer perceptron neural network.

A major advancement of the perceptron model was its ability to learn accurate weights from training datasets under supervised learning. The simple elegance of the learning algorithm is found in its ability to predict an output and adjust a bias factor (*b*) according to how the output matches the prediction. If an input class γ matches the predicted $\hat{y}$, no adjustment is made to the input weight or the bias factor. However, if an input class γ does not match the predicted $\hat{y}$, the bias factor is automatically updated to multiply the input weight to match the predicted value. Additionally, the threshold for propagation (theta) is not hand coded as in the McCulloch-Pitts artificial neuron model. Instead, theta is also learned by being included as a synaptic input. A depiction of a perceptron including the net input and activation function is shown in Figure 1A.

The perceptron model is single-layer in nature and can only accommodate linearly separated data. A neural network that consists of only a single-layer includes only one set of input nodes. Output nodes may exist singularly or there can be several. The dual function of output node(s) also includes the duties of receiving node(s). Due to its single-layer architecture, the perceptron model is limited in scope, since it cannot solve the XOR problem. In order to implement an XOR solution, a multilayer neural network is needed to work with non-linearly separated data. There have been several proposed solutions for the XOR problem using multilayered perceptron (MLP) networks (Yanling et al., 2002; Yang et al., 2011; Singh, 2016; Samir et al., 2017).

A MLP neural network consists of perceptrons organized into an input layer, at least one hidden layer, and an output layer. A deep neural network requires more than one hidden layer. A MLP that only has one hidden layer is sometimes referred to as a “vanilla” neural network. Rumelhart et al.’s (1986) seminal paper explained the fundamental learning processes of MLPs, including the feedforward pass and the particularly significant backpropagation step (Rumelhart et al., 1986). The feedforward nature of the MLP ensures that information is passed through the network in only one direction: from the input layer, through the hidden layers and finally through the output layer. There is no circular movement of data passage. The subsequent backpropagation process involves sending information from a forward pass back throughout the network, from the output to the input layer, while adjusting the bias and weight parameters. The purpose of backpropagation is to minimize the cost function by reducing the difference between the anticipated output value and the actual output value. It does so by calculating the local gradient via the chain rule to then correct the synaptic weights and biases (Svozil et al., 1997).

Although the utility of MLPs in computer vision was an important step forward, modern computational demands have shown MLPs to be limited. MLPs are fully connected networks. Therefore, all layers of perceptrons are fully connected to all other layers of perceptrons (Figure 1B). Since MLPs are fully connected, their time and space complexity grow exponentially with every additional layer in the network, making them vulnerable to inefficiency and impracticality for complicated tasks (Mühlenbein, 1990). Additionally, each perceptron receives only one unit of input, such as an image pixel and associated weight. Therefore, the need for processing large images renders MLPs a non-optimal option. MLPs also require flattened vector inputs for image processing, so spatial information becomes lost (Feng et al., 2019). Finally, MLPs also run the risk of overfitting training data, leading to poor generalizability (Caruana et al., 2001). These shortcomings led to the development of higher-complexity methodologies.

### Convolutional Neural Networks

Convolutional neural networks (CNNs) have emerged as the standard tool for computerized image classification (Rawat and Wang, 2017; Abbas et al., 2021). Additionally, the application of CNNs has extended to face detection, facial expression recognition and speech recognition (Wang et al., 2020). Similar to MLPs, CNNs can receive an input image, learn weights and biases to then differentiate between images. However, unlike MLPs, CNNs can fully grasp the spatial and temporal dependencies of an image without requiring flattening. Furthermore, CNN architecture allows them to make accurate assumptions about specific, relevant features and patterns within images without prior knowledge (Jarrett et al., 2009). Training a CNN is more efficient than training a MLP because layers are sparsely connected, weights are smaller and shared among blocks of features, rather than between individual pixels. Furthermore, CNNs demonstrate robust applications due to their impressive generalizing ability. The first CNN model was proposed as the neocognitron (Fukushima and Miyake, 1982). Since the neocognitron, there has been tremendous advancements in CNN application and methodology research (Wu et al., 2020). Some notable advancements include the LeNet-5 and AlexNet models (LeCun et al., 1989; Krizhevsky et al., 2012).

The network layers of a CNN include the input layer, the convolution layer(s), the pooling layer(s) and the fully connected layer. The process of convolution is traditionally a linear operation of feature extraction. However, non-linear convolution has also been implemented (Zoumpourlis et al., 2017; Marsi et al., 2021). Convolutional layers include at least one kernel, or filter, which is a learnable parameter of the network. The kernel(s) compute feature maps corresponding to the receptive field with shared weights of a block of pixels (Agrawal and Mittal, 2020). Weight sharing reduces the number of training parameters to help CNNs to avoid overfitting and boost generalizability. The kernel operations compute the element-wise product of the two tensors in the convolutional layer. The output of convolving an input block with a kernel is a feature map; thus, the output of a convolutional layer is a number of kernelized images (*I*) equivalent to the number of kernels (*K*) with shape (*I*_*h*_−*K*_*h*_ + 1,*I*_*w*_−*K*_*w*_ + 1,*I*_*d*_−*K*_*d*_ + 1) for a single-channel (e.g., grayscale) image. The kernel **K** output **F** with dimensions (**i**,**j**,**k**) of a 3D image **I** with dimensions (**p**,**q**,**r**) corresponds to:

F⁢(i,j,k)=(K*I)⁢(i,j,k)

(1) $$=\sum_{p}\sum_{q}\sum_{r}I\,\left(i-p,j-q,k-r\right)\,K\,\left(p,q,r\right)$$

Convolutional layers contain hyperparameters that can be optimized, such as padding (Dwarampudi and Reddy, 2019). A major advancement of CNNs over MLPs is that they reduce image complexity for faster processing. Because the convolutional layers progressively reduce the original image’s size, pixels or voxels on the borders get lost. This can become an issue in very deep networks because it may lead to important image features being lost during training. The most common solution to the border loss issue is to use padding. Setting the padding hyperparameter corresponds to adding extra zero value pixels or voxels around the borders of the input image. Padding (*P*) changes the shape of the output of the convolutional layer, which becomes (*I*_*h*_−*K*_*h*_ + *P*_*h*_ + 1,*I*_*w*_−*K*_*w*_ + *P*_*w*_ + 1,*I*_*d*_−*K*_*d*_ + *P*_*d*_ + 1) (Zhang et al., 2021).

Another hyperparameter for the convolutional layer is stride. When stride is set to one, the computation on Eq. 1 is done for all pixels in the image block and the kernel is considered to be non-strided. Increasing stride to two means that convolution will be applied to every other pixel in the image block. Tuning the stride length of the kernel to broader units greatly increases computational efficiency in training the network (Krizhevsky et al., 2012; Zeiler and Fergus, 2014). Kernels move left-to-right and top-to-bottom along an image matrix according to the stride value until the entire image is crossed. In an architecture that includes several convolutional layers, the first convolutional layers extract low-level features of an image, whereas deeper layers extract high-level features.

To further reduce dimensionality, pooling layers are added after the convolutional layers. Pooling is used to extract important features and further down-sample the input image size. Pooling is a matrix summarization technique where a filter with size (*p*,*q*,*r*) is chosen and the input image is traversed in a sliding window fashion to reduce each block of that size. In other words, a cluster of output points, whose size depends on a tunable stride hyperparameter, is summarized into one neuron in the next layer. To prevent down-sampling different input spaces into the same information, these layers perform max or average pooling. With the max pooling approach, all sub-matrices in the input space with the size of the chosen stride are checked and the maximum value of each is chosen. With the average pooling approach, the average is computed and chosen as the summarized value (Persello and Stein, 2017).

In most CNN architectures, the output of the convolutional layer is passed through a non-linear activation function as in a traditional neural network. Common choices for activation are sigmoid functions, such as the logistic or the hyperbolic tangent function. LeNet-5 used a sigmoid logistic function as its activation function after the pooling layers (LeCun et al., 1998). Recently, the rectified linear unit activation function (ReLU) became standard in neural networks, including CNNs, because of reduced likelihood of having vanishing gradients during backpropagation, as well as not requiring input normalization to prevent saturation (Nair and Hinton, 2010; Ramachandran et al., 2017). These properties make the ReLU faster for deep network training. AlexNet used the ReLU in its convolutional and fully connected layers and showed that a 0.25 error rate was reached six times faster than with the hyperbolic tangent on the CIFAR-10 dataset (Krizhevsky et al., 2012). Occasionally, although less likely than with sigmoid functions, ReLU may lead to the vanishing gradient problem due to leading to sparsity. To circumvent this, modified versions, such as leaky ReLU, and parametric ReLU can be used (Jiang and Cheng, 2019).

**FIGURE 2:**
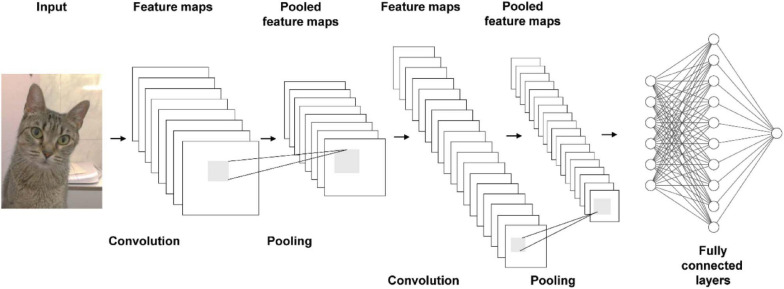
General architecture of a CNN.

Classification of the reduced input space is done by the last layers of the CNN, which correspond to dense layers in a fully connected feedforward network as described in the previous section. Several variations of the classic architecture of the CNN have been introduced over the last decade to tackle issues, such as overfitting and computational cost: GoogLeNet, ResNet, Inception-4, and VGG-16 (Simonyan and Zisserman, 2015; Szegedy et al., 2015, 2017; He et al., 2016). These architectures have been established as standard models for image classification due to their great success on addressing this task, with thousands of papers adapting them for specific problems (Rawat and Wang, 2017). The general architecture for a CNN is presented in Figure 2.

### Generative Adversarial Networks

**FIGURE 3:**
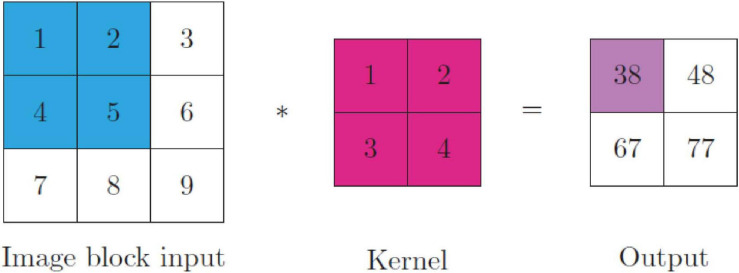
The convolution operation (*) between an image input and a kernel. The element-wise product of the image block (blue) with the kernel (magenta) is calculated and added together (purple). All blocks are convolved with the kernel to generate an output of shape *I*_*h*_−*K*_*h*_ + 1,*I*_*w*_−*K*_*w*_ + 1.

The GAN were first introduced in 2014 and is an adversarial framework where two neural networks, generative and discriminative, compete against each other (Goodfellow et al., 2014). The generative network *G* generates synthetic examples derived from a random distribution, and the discriminative network *D* evaluates whether the provided example is real or modeled input (Figure 3). Consider a data input space *x*. The generator network *G*, an MLP, learns a data distribution *p*_*G*_(*x*) by mapping a prior distribution *p*_*z*_(*z*) on a random noise variable through its parameters. The discriminator *D*, also an MLP, is trained to maximize the probability of correctly classifying an input as being derived from *x* or *p*_*G*_(*x*), that is, it aims to maximize *log*⁡*D*(*x*) + log(1−*D*(*G*(*z*))) where *D*(*x*) is the probability of the example being real and *D*(*G*(*z*)) the probability of having been generated by *G*. Network *G* is simultaneously trained to minimize the cross-entropy loss function given by log(1−*D*(*G*(*z*))) and thus progressively learn to generate examples with low probability of being classified as synthetic by *D*. This is thus a minimax optimization game between *G* and *D* with value function *V*(*G*,*D*) defined by:

$$\underset{G}{m\,i\,n}\underset{D}{m\,a\,x}V\,\left(G,D\right)={𝔼}_{x\,p\,d\,a\,t\,a\,\left(x\right)}\,\left[l\,o\,g\,D\,\left(x\right)\right]$$

(2) $$+{𝔼}_{z\,p\,\left(z\right)}\,\left[\text{log}\,\left(1-D\,\left(G\,\left(z\right)\right)\right)\right]$$

This minimax game has its global optimum at *p*_*g*_ = *p*_*data*_ when *G*’s distribution for *x* equals the distribution of *x* (Gonog and Zhou, 2019). Training of a GAN consists of sampling minibatches of real data examples and of examples derived from *G*’s *p*_*z*_(*z*) distribution and updating *D*’s parameters by stochastic gradient ascent of *log*⁡*D*(*x*) + log(1−*D*(*G*(*z*))), followed by sampling minibatches of data points from *p*_*z*_(*z*) and updating *G* through stochastic gradient descent of log(1−*D*(*G*(*z*))) until an optimum is reached (Goodfellow et al., 2014). In practice, however, the generator’s loss function quickly saturates and becomes more efficient to train *G* to maximize log(*D*(*G*(*z*))). However, instead, *G* seeks to maximize the probability of examples being classified as real rather than minimize the probability that they are synthetic (Goodfellow et al., 2014). Other loss functions for GANs have been proposed. For example, Mao et al., 2016 proposed the least squares GAN, where the loss function is the mean squared error of the predictions; because least squares penalize errors more strongly, it is less likely to lead to vanishing gradient issues and was found to perform better. Other solutions have been proposed, such as the Wasserstein GAN (Arjovsky et al., 2017) and the DRAGAN (Kodali et al., 2017). Lucic et al. (2017) showed that these variations achieved similar performance on several benchmark datasets, such as the MNIST, CIFAR, and CELEBA (LeCun et al., 1998; Krizhevsky and Hinton, 2009; Liu et al., 2014).

**FIGURE 4:**
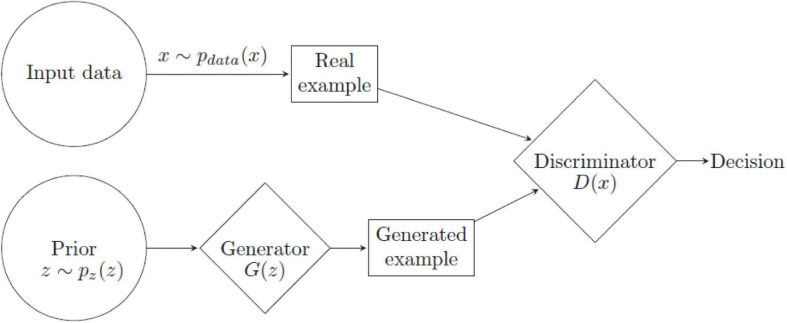
Real input datapoints *x* and examples generated from a prior distribution by network *G* are fed to discriminator *D* for classification.

Generative adversarial networks (GANs) are popular in the computer vision (CV) field, particularly for data augmentation. Popular architectures are deep convolutional GANs (DCGANs) (Radford et al., 2016), conditional GANs (Mirza and Osindero, 2014), pix2pix (Isola et al., 2016), and CycleGAN (Zhu et al., 2017). DCGANs are GANs where the generator and discriminator networks are all-convolutional networks that learn from real data to subsequently generate synthetic examples for image classification (Figure 4).

### Ensemble Learning

Ensemble Learning (EL) has emerged as a popular solution in CV. EL provides high complexity with a training process that incorporates separate classifiers that are learning from distinct data subsets to be aggregated for final classification. The main modalities of EL are bagging, boosting, stacking, and mixture of experts. Bagging, “Bootstrap Aggregating,” trains different classifiers on bootstrapped samples in combination (Breiman, 1996). Briefly, *n* training sets are generated from the original dataset through uniform sampling with replacement, to ensure that the samples remain independent and maintain a distribution similar to the original dataset. Next, *n* models are trained, one per sample, and combined by voting. Bagging can prevent overfitting and reduce variance in high-variance datasets (Breiman, 1996; Bühlmann and Yu, 2002). Bühlmann and Yu (2002) showed that in hard classification problems that lead to instability (defined as small changes in the data leading to wildly varying predictions), bagging smooths the decision and yields smaller variances. Bagging of multiple MLPs has been shown to be successful at increasing performance, relative to a single MLP (Gençay and Qi, 2001; Ha et al., 2005). Boosting, specifically adaptive boosting (Adaboost), is a meta-algorithm that consists of weak classifiers being iteratively trained on a dataset and added together in a weighed manner dependent on their classification accuracy (Schapire, 1990; Freund and Schapire, 1999). These weak classifiers learn from each other, in the sense that incorrectly classified examples are penalized with a weight. The following weak classifier will therefore give more importance to examples misclassified by previous learners to ensure that performance progressively increases (Freund and Schapire, 1999). While originally developed to boost the performance of decision trees, boosting has been shown to also increase performance of DL models. For example, Moghimi et al. (2016) proposed a new algorithm incorporating boosting with CNNs (BoostCNN). Han et al. (2016) proposed in 2016 an incremental boosting approach for CNNs to avoid overfitting in predicting facial expression. The architecture incorporates incrementally updated Adaboost layers that select neurons from the previous layer to learn weights and was shown to improve model metrics compared to traditional CNNs on benchmark datasets.

Stacking is another ensemble meta-algorithm that combines predictions from different models (Wolpert, 1992). In a stacking architecture, the main model learns how to combine the best predictions from other contributing models that, unlike in bagging and boosting approaches, can be based on different algorithms. Essentially, a meta-classifier learns whether the data was correctly classified by smaller classifiers trained on bootstrapped samples. Deng et al. (2012) proposed a method based on stacking to upscale training of complex neural networks. Finally, the mixture of experts is a methodology in which several models are trained on the same data and their outputs are gated through a network that linearly combines them to obtain a final classification (Jacobs et al., 1991). Shazeer et al. (2017) used this approach to combine thousands of MLPs to achieve strong performance with low computational costs.

## Methodologies for AD Imaging Classification

### CNN

Computer vision (CV) models based on CNNs have become popular for solving the Alzheimer’s disease classification problem. Frequently, medical imaging processing requires preprocessing to capture regions of interest (ROI) (Elsayed et al., 2012). This can be done manually or using signal processing techniques, such as the Hough transform (Duda and Hart, 1972) and the scale-invariant feature transform (SIFT) (Lowe, 1999). ROI-based approaches can be implemented in a CNN, such as the region-based CNN (R-CNN) (Girshick et al., 2014; Girshick, 2015). Mercan et al. (2019) also proposed a patch-level framework where a VGG-16 CNN is trained on patches extracted from ROIs and learns important features from weighted average pooling of its features. This approach was shown to be successful in detecting cancerous abnormalities in breast histopathology images (Mercan et al., 2019). However, one of the advantages of CNNs over other neural network architectures is that it does not require manual extraction of relevant features based on prior knowledge.

Modeling for AD imaging classification can be either binary (e.g., cases vs. control) or multiclass [e.g., cognitively normal (CN), mild cognitive impairment (MCI), early (EAD), and late onset AD (LAD)]. CNN models, including several of the standard neural network algorithms, such as GoogLeNet and ResNet are proven effective at deep multiclassification analysis in medical imaging (Li et al., 2014; He et al., 2016; Szegedy et al., 2017; Khan and Yong, 2018; Wang et al., 2018; Alsharman and Jawarneh, 2020). For accurate medical imaging recognition and/or classification, CNNs must be deep enough and able to extract features from the data at varying scales. Although the architecture of CNNs makes them more appropriate for image analysis, too many suffer from overfitting and exponentially increasing computing burden as they become large enough. Lin et al. (2014) introduced the network-in-network concept, which consists in adding small nets within a larger CNN to allow for data abstraction within each receptive field.

The GoogLeNet leveraged this concept and introduced inception modules that use multiple convolution filters within the same layer to allow a deeper architecture, and then concatenates the results. Furthermore, an auxiliary classifier was added to tackle the vanishing gradient problem due to the network’s depth, as well as prevent overfitting by adding regularization parameters.

The GoogLeNet architecture has achieved success in classification of AD imaging data (Farooq et al., 2017). Prakash et al. (2019) tested the GoogLeNet, AlexNet, and VGG-16 networks for classification of the ADNI MRI dataset in CN, MCI, and AD to show that GoogLeNet achieves 99.84% accuracy in training and 98.25% in the test set, higher than the remaining architectures. These results illustrate the advantage of the GoogLeNet architecture in preventing overfitting by using auxiliary classifiers, compared to other models, such as AlexNet and VGG-16.

ResNet is an additional successful variant of the classical CNN (He et al., 2016). The hallmarks of the ResNet model include the incorporation of residual mapping rather than unreferenced as well as the ability to skip layers in the network to create “shortcut connections.” In this way, residual learning converges faster and addresses the degradation problem, where accuracy saturates and then degrades as the network grows deeper.

Farooq et al. (2017) investigated the performance of GoogLeNet and two ResNet models, with 18 and 152 layers, in solving a multiclass analysis of AD and MCI using the ADNI MRI data. The authors developed a four-way classifier to classify AD, MCI, late MCI, and controls. Data augmentation was performed on these images by flipping them along the horizontal axis as the left and right brain region are symmetrical. The proposed approach for 4-way classification achieved accuracies of 98.88, 98.01, and 98.14% using the GoogleNet, ResNet-18, and ResNet-152 pre-trained networks, respectively. All three architectures performed better than other models that are proposed to classify AD MRI data, such as the stacked auto-enconder (SAE) models (Gupta et al., 2013; Ferri et al., 2021). Furthermore, Valliani and Soni (2017) proposed a pre-trained deep ResNet to classify AD MRI imaging in order to demonstrate that training on biomedical imaging was not necessary for the task and achieved modest accuracy. Lastly, Fuad et al. (2021) performed a comprehensive longitudinal comparison of different architectures for the classification of brain cancers revealing an increase of roughly 5% in classification accuracy over the past 5 years (Table 1).

**TABLE 1:** Comparison of recent architectures used for classification of brain cancer (adapted from Fuad et al., 2021).

**Authors**	**Method**	**Accuracy (%)**
Cheng et al., 2015	Intensity histogram	87.5
	GLCM	89.7
	BOW	91.3
Paul et al., 2017	Deep learning CNN	91.4
Afshar et al., 2018	CapstNets	90.9
Fuad et al., 2021	AlexNet	94.6
	GoogleNet	92.0

3D CNN architecture has been utilized to take whole brain MRI scan as input and output the classification results. Payan and Montana (2015) described a framework based on unsupervised training of a sparse auto-encoder to learn convolutional filters to then use in a 3D CNN for three-way classification. The authors posited that the use of a sparse auto-encoder for filter learning could be advantageous to control for underlying factors responsible for MRI data variability. The learned filters were used as parameters in a 3D CNN architecture, whose performance was tested against a 2D one. The authors showed that the 3D approach marginally increases accuracy of three-way classification due to it capturing 3D patterns in the data. Feng et al. (2020) described an approach where 3D CNNs were used for AD MRI image classification in conjunction with 2D MRI slice scans to overcome their inability to provide contextual information on their connectedness. The 3D-CNN architecture contained stacks of batch normalization (BN) and ReLU activation function. A max pooling layer was then used to extract features from the volumes and to reduce the dimensionality of the data, followed by a dropout layer. Despite the increased computational cost of using 3D convolution as an extension of 2D convolution, accuracy for three-way classification of AD, MCI, and controls improved from 82.57 to 89.76% for regularization. The authors also used a 3D-CNN-support vector machine (SVM) classifier. The SVM classifier did not add any time complexity but was able to improve accuracy to 95.74%which was statistically better than the other two approaches.

### GANs

Generative adversarial networks (GANs) are used in the detection of AD to enhance brain MRI scans or to predict whole brain image structure at a future point in time. Using GANs for forecasting brain alterations assists in precise early neurodegenerative disease detection. Although labeled training data is expensive to find in AD imaging datasets, several GAN architectures have been developed to reduce this computational burden through augmenting data, extending training datasets and sending them to deep learning classifiers. An impressive example of GAN synthetic data augmentation is provided by Kazuhiro et al. (2018), who were able to use almost one hundred T1-weighted MRIs from 30 healthy controls and 33 stroke patients with DCGANs to generate synthetic MRI images. These generated images were unable to be detected as fake by radiologists and neuroradiologists.

In addition to synthetic data augmentation, GAN has also been proven useful in enhancing the quality of MRIs, which has led to better performance in AD classification. The diagnostic quality of MRI images for AD is dependent on the signal-to-noise ratio (SNR), which is influenced by the instrument’s parameters (e.g., magnetic strength). The performance of AD classification models is highly correlated to the advancements of the scanners. Zhou et al. (2021) recently explored the relationship between GAN performance using T1-weighted MRIs of various quality and AD classification accuracy. Both 1.5-Tesla (1.5-T) and 3-Tesla (3-T) scans were produced during the same patient visit and were available for this study. 3-T images are constructed using a twice-than-normal strength magnet and therefore offer a much clearer image with half of the noise-to-signal ratio. The authors first used a GAN to generate synthetic images, referred to as a 3T^∗^ images, based on the 1.5-T scans. Subsequently, a discriminator was used to analyze the similarities and differences between the 3T^∗^ images and the same-patient 3-T scans. The 3T^∗^ images were then used to train a fully convolutional network (FCN) to identify AD verses control cases. Cross-entropy loss was reduced through simultaneous GAN and classifier loss minimization. This GAN-based deep learning strategy was able to identify how to improve the 1.5-T images to meet or exceed the quality seen in the 3-T images using a GAN approach.

There are clinical advantages to combining PET scans with MRI scans. MRI scans allows clinicians to observe soft tissue contrast, whereas PET scans allow clinicians to observe metabolic function at a cellular level. Multimodal assessment can increase diagnostic power, which has been shown to be the case in AD (Fang et al., 2020). However, PET scans require the use of radioactive tracer dye, which may not be always feasible due to allergic reactions to the iodine tracer, other contra-indicated health conditions or simply cost and time restrictions.

Lin et al. (2021) used a 3D reversible generative adversarial network (RevGAN) to generate missing PET scans based on what would have been complimentary MRI images from the same AD patient. RevGAN consists of one reversible generator and two discriminators. The generator has three components: encoder, invertible core, and decoder. Each of them consists of a series of blocks of convolution, normalization, and a ReLU layer. After RevGAN use, a 3D-CNN was able to be successfully employed to use multi-modal input to make a distinction between AD and control images. This method was confirmed against images from the ADNI database.

### EL

Ensemble models based on CNNs have recently been explored for image classification in AD. Zheng et al. (2018) proposed an ensemble of AlexNets to classify PET imaging data of patient brains as normal or affected by AD, and further distinguish between stages of MCI. Specifically, the authors adopted a patch-based approach for feature extraction by using the Automated Anatomical Labeling software to segment the PET brain images into distinct neuroanatomical regions. This strategy was adopted since AD-associated neurodegeneration affects certain regions of the brain disproportionately (Lam et al., 2013). Moreover, it has the advantage of not requiring manual annotation. Each set of image patches, representing a different brain region, was then fed to an AlexNet CNN to be classified as healthy or affected by AD, or by MCI severity in affected patients. The best performing models were then chosen by majority voting. By adopting this approach, the authors achieved an accuracy of 91% for healthy vs. AD classification and 85% for mild MCI vs. severe MCI, an improvement in performance relatively to other deep learning methodologies (Karwath et al., 2017; Valliani and Soni, 2017). Tanveer et al. (2021) developed a novel ensemble model, DTE, which utilizes a combination of deep learning and transfer learning, and ensemble learning. DTE was tested on a large ADNI dataset, which showed that DTE achieved a maximum classification accuracy of 99.09% for NC vs. AD and 98.71% for MCI vs. AD classification. When DTE was tested on a small ADNI dataset, DTE achieved a maximum classification accuracy of 85% for NC vs. AD.

Another methodology was developed by Islam and Zhang based on a bucket of six CNN models with distinct architectures trained on the OASIS dataset containing MRI imaging data for healthy individuals and AD patients (Marcus et al., 2010; Islam and Zhang, 2018). The authors tested different ensembles of the six models, with the best performing one consisting of three CNNs with alternating dense layer blocks and convolution-pooling blocks. The ensemble achieved an accuracy of 93%, outperforming other architectures, such as ResNet, ADNet, and Inception-v4 as tested by the authors on the same dataset. Additional CNN ensemble models have been proposed for this task (Wang H. et al., 2019; Pan et al., 2020). An et al. (2020) developed DELearning, a three-layer framework for AD classification that uses the deep learning approach to ensemble at each layer to integrate multisource data. Using clinical data from NACC UDS, the authors tested DELearning against six other EL methods: LogitBoost, Bagging, Random Forest, AdaBoostM1, Stacking, and Vote. Results showed the DELearning was able to outperform all six methods in terms of precision, recall, accuracy, and F1-measured. DELearning showed a 3% increase in recall and a 4% increase in accuracy compared to the other methods.

Approaches for multi-modal imaging data classification have also been developed. Fang et al. (2020) proposed an ensemble approach for multi-modal data that takes advantage of a deep CNN for automated feature extraction followed by classification with Adaboost. Specifically, the authors built a stack of three deep CNNs (GoogLeNet, ResNet, and DenseNet) that learn hierarchical representations from the MRI and PET modalities separately and compute a classification score for each example based on each data type. The predictions from the two data modalities are then combined through Adaboost. This ensemble achieved an average accuracy of 93% for both healthy vs. AD and healthy vs. MCI. This methodology facilitates feature extraction through abstraction, given that human annotation of ROI is not required.

In addition to CNNs, other model architectures have been proposed with ensemble approaches for AD imaging classification: hierarchical ensemble learning with deep neural net (Wang R. et al., 2019), learning-using-privileged-information (LUPI) algorithms (Zheng et al., 2017), sparse regression models (Suk et al., 2017), and instance transfer learning (Tan et al., 2018).

## Conclusion

**FIGURE 5:**
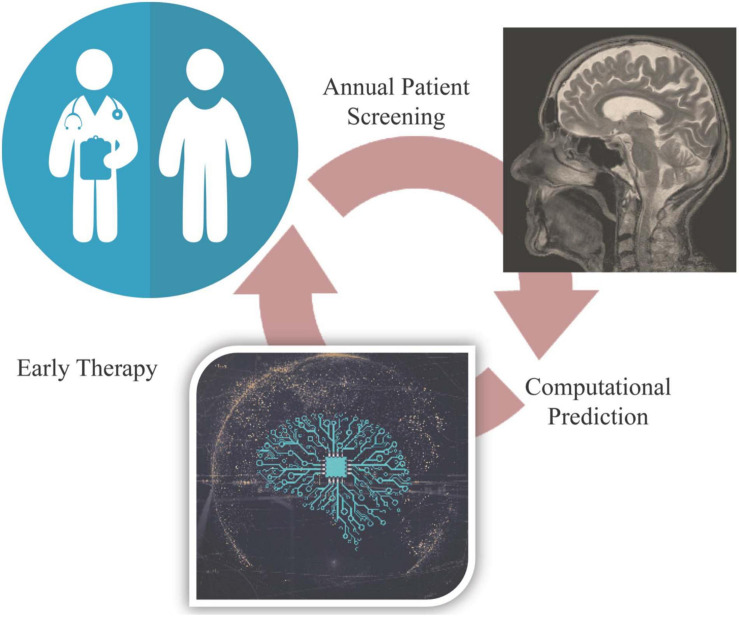
Proposed approach for computer vision-assisted early diagnosis.

Alzheimer’s disease continues to be an incurable pandemic. Advanced methods to improve disease detection are crucial. We propose a computer vision assisted approach for detection before clinical symptom onset (Figure 5). The rise in the power of computational models is for the first-time allowing scientists to analyze and extract meaningful clinical insights from previously untouched massive datasets. It is imperative that the scientific community continue to adapt and move forward with interdisciplinary approaches to tackle the world’s greatest unknowns, including neurodegenerative disorders.

## Author Contributions

RL, MFS, AI, and SM were responsible for the initial drafting of the manuscript. RL, BW, MFS, AI, NS, AG, and SM were responsible for satisfying the reviewers and approve of the final work.

## Conflict of Interest

MFS, AI, AG, RL, and SM are current employees of Pluripotent Diagnostics Corp. This study received funding from Pluripotent Diagnostics Corp. The funder was involved in manuscript preparation and decision to publish. The remaining author declares that the research was conducted in the absence of any commercial or financial relationships that could be construed as a potential conflict of interest.

## Publisher’s Note

All claims expressed in this article are solely those of the authors and do not necessarily represent those of their affiliated organizations, or those of the publisher, the editors and the reviewers. Any product that may be evaluated in this article, or claim that may be made by its manufacturer, is not guaranteed or endorsed by the publisher.
